# Extracts from a Letter from E. L. Holmes, M. D., of Chicago

**Published:** 1862-11

**Authors:** 


### FOREIGN CORRESPONDENCE.

### EXTRACTS FROM A LETTER FROM E. L. HOLMES, M. D., OF CHICAGO.

VIENNA, July 27, 1862.

I have now been in this city nearly six weeks, and have spent several hours daily in the hospital. I am more convinced than ever, that in many respects, Vienna offers the medical student better facilities for the study of clinical medicine, than can be found in any other city in the world. I express this opinion not only from my own personal experience and observation, but also from the testimony of medical men from nearly every country of Europe and America, whom I have met here and in other German cities. Practitioners who have spent months in London, Paris and Berlin, whose opinion is worthy of respect, have assured me that for the study of general medicine and surgery, they believe Vienna is superior to all other cities of Europe. This superiority consists not in the fact that there are more patients and better instructors in Vienna, but that here everything is arranged especially for the convenience of the student, so that he can attend some clinic at almost any hour of the day, and can also take special instruction in the diagnosis and treatment of the diseases of nearly every organ of the body.

And yet I may here say that American students, who have improved all the advantages offered them in our large cities, and have had the opportunity of spending a year or two in a good hospital, as assistants, need not fear a comparison with the great majority of medical students who are graduated at the European universities. But comparatively few of our students in America have the privilege of residing as assistants in hospitals, or of *spending several hours daily* in the clinical study of disease.

The present general Hospital—Allgemeinenkrankenhaus—was founded in the year 1784 by the Emperor Joseph II. It is situated in one of the municipal divisions of the city, near the glacis or open space which serves as a park for the people of Vienna. Although surrounded on all sides by dwellings, the proximity to this open space and the constant change of air produced by the strong winds which continually blow from the distant mountains render the locality of the hospital quite healthy. The grounds of the hospital, irregular in shape and extending over many acres, are surrounded by a series of two-story buildings. On one street the hospital is more than one thousand feet long, and on another running at right angles to the first, more than six hundred feet. The other two sides of the grounds are quite irregular. This large enclosure is subdivided into ten large courts, by series of buildings running at right angles with each other. These courts communicate with each other by means of large arches formed through the buildings and are laid out in pleasant walks and greens, and ornamented with good shade trees, shrubbery and fountains, thus affording suitable promenades for convalescent patients. The largest court, into which one enters from the main street under a high archway, contains several acres, ornamented as I have described, and in itself is a proof of the taste and humanity of the founders of the hospital. On the side of this court, opposite to the entrance, is the hospital chapel. On the left hand side is a building containing the offices of the superintendent and his assistants and several wards for patients, in two of which Scoda gives his clinical lectures.

These buildings are almost wholly occupied by patients, certain portions being reserved for residences of visiting surgeons and physicians, their assistants and others employed in the hospital; and also for store rooms for provisions, linen bedding, kitchen, etc.

The hospital is furnished with a good supply of drugs and medicines, which are dispensed by the hospital apothecary.

I have been informed that the present capacity of the hospital is sufficient for two thousand patients, although a much greater number can be accommodated in emergencies. In addition to the patients there are a large number of persons constantly employed in nursing, washing linen and repairing and cleaning the building of the hospital.

The buildings are very simple, plain structures, without any of the so-called modern improvements in the mode of ventilating, heating or supplying water. There are generally no means of ventilating the wards except from the windows on each side. As each ward is usually provided with many windows, the change of air is readily effected, although the beds are placed at a distance of only two feet and a half apart.

Some of the wards in the lower stories, however, are dark, damp, and not well ventilated.

Upon the whole the hospital, with its bedding and clothing for the patients, is kept as clean as usual in such large public institutions. In several points relating to sanitary arrangements the hospital is inferior to the new hospitals of Hamburg, Berlin, Paris and other cities that I have visited.

Back of the hospital is the fine new building in which is the splendid pathological museum of Rokitansky, the dissecting rooms and the "dead house." The dead are left but an hour or two in the wards, when they are removed to a room in this building and placed upon beds, over which are machines connected by a cord with the hands of each corpse. The least movement of the hand connected with this cord moves a spring and rings a bell for the watcher. This precaution is taken to prevent premature interments. I was informed, however, that in no instance had a case been known in which a body supposed to be dead had given evidence of life by means of this contrivance.

The number of patients treated annually in this hospital is about 24,000. As the sick are brought to the hospital they are classified according to their diseases. The student will find wards filled respectively with patients affected with diseases of the eye, skin, chest, with syphilis and surgical and medical diseases. There are wards for lying-in women and for female patients affected with diseases peculiar to their sex. When we consider the number of patients constantly under treatment and the number annually received, we can readily understand what facilities the student may find for the study of every form of disease.

I intended to give an account of the different clinics and of the manner in which medical instruction is given in this hospital, but I must defer it to another time.

Allow me to say that I promised at the close of my last letter to speak of the "clinics," and not of the "churches," of Vienna, as printed.

